# Effect of Multimeric Structure of CaMKII in the GluN2B-Mediated Modulation of Kinetic Parameters of ATP

**DOI:** 10.1371/journal.pone.0045064

**Published:** 2012-09-18

**Authors:** John Cheriyan, Archana G. Mohanan, Pradeep K. Kurup, Madhavan Mayadevi, Ramakrishnapillai Vyomakesannair Omkumar

**Affiliations:** Molecular Neurobiology Division, Rajiv Gandhi Centre for Biotechnology, Thycaud, Thiruvananthapuram, Kerala, India; Universidade Federal do Rio de Janeiro, Brazil

## Abstract

Interaction of GluN2B subunit of N-methyl-D-aspartate receptor with calcium/calmodulin dependent protein kinase II (CaMKII) is critical for the induction of long term potentiation at hippocampal CA3-CA1 synapses. We have previously reported that CaMKII binding to GluN2B increases its affinity but abolishes the cooperativity for ATP. In the present study, we demonstrate that the reduction in S_0.5_ for ATP of an individual CaMKII subunit seems to be directly induced by the binding of GluN2B to the same subunit, while any GluN2B induced effects on the cooperativity and maximal velocity would additionally require the CaMKII holoenzyme structure. We measured the apparent kinetic parameters for ATP using an association domain truncated monomeric CaMKII and a heteromultimeric CaMKII (having subunits that are either GluN2B binding defective or ATP binding defective), in the presence of GluN2A or GluN2B substrates. The S_0.5_ value for ATP of monomeric CaMKII is reduced ∼ 3 fold by the presence of GluN2B suggesting that the induced change in affinity for ATP is independent of the holoenzyme structure. The heteromultimeric mutant of CaMKII, did not exhibit cooperativity of ATP binding probably because of the interspersing of ATP binding defective subunits in the holoenzyme. In contrast to the wild type holoenzyme, presence of GluN2B increased the V_max_ of monomeric CaMKII which resulted in an approximately 4.0 fold increase in the apparent catalytic constant (V_max_/S_0.5_) as compared to GluN2A. The kinetic parameter values of the heteromultimeric CaMKII for ATP, on the other hand, did not show any significant difference between the phosphorylation of GluN2B and GluN2A suggesting that modulation requires binding of GluN2B to the same subunit. Overall, our present study provides insights into the role of multimeric structure of CaMKII in GluN2B-mediated regulation.

## Introduction

Calcium/calmodulin dependent protein kinase II (CaMKII) is a kinase of broad substrate specificity that is abundant in the brain and is involved in many aspects of cellular function such as the regulation of metabolism, channel function, neurotransmitter synthesis and release, transcription, cytoskeletal organization, intracellular calcium homeostasis, long-term potentiation (LTP) and neuronal memory [1], [2]. Its unique molecular structure and activity regulation led investigators to speculate about its role in learning and memory [3]. It has now been convincingly shown that CaMKII and N-methyl-D-aspartate receptor (NMDAR) are involved in LTP at the excitatory glutamatergic synapses [4], [5], [6]. In the postsynaptic compartment, Ca^2+^ influx through NMDAR activates CaMKII, following which, it translocates from cytosol to postsynaptic density (PSD) and binds to NMDAR subunit 2B (GluN2B) [7], [8], [9]. GluN2B can bind to CaMKII via a non-catalytic site, enabling the enzyme to remain autonomously active [10]. The binding is also bi-directionally modulated by the presence of nucleotides and phosphorylation [11], [12]. The activation of CaMKII drives a series of biochemical pathways that leads to LTP mediated by increase in the activity of synaptic α-amino-3-hydroxy-5-methyl-4-isoxazolepropionic acid receptors (AMPAR) [4]. The disruption of CaMKII-GluN2B interaction has been shown to produce deficits in hippocampal LTP and spatial learning [13]. In addition to functioning as an anchoring point at the postsynaptic density, GluN2B can also modulate the activity of CaMKII [10], [11], [14], [15], [16]. If this binding is disrupted, phosphorylation of the GluA1 subunit of AMPAR by CaMKII (which enhances AMPAR conductance) is impaired and LTP and spatial learning are affected [13], [17], [18], [19]. Earlier reports had suggested that the multimeric structure of CaMKII is required for its binding to GluN2B although later studies showed interaction of GluN2B to truncated CaMKII that exist as monomers [20], [21], [22]. Our group has reported that binding of GluN2B to CaMKII modulates the affinity and kinetic parameters of CaMKII for ATP [14], [15].

In the present study, an attempt was made to investigate whether the holoenzyme structure of CaMKII had any role in the regulation of ATP kinetics induced by GluN2B. For this, we used an association domain truncated mutant of CaMKII (Δ317-α-CaMKII), which is known to be monomeric [20], [22] and a heteromeric CaMKII mutant consisting of subunits defective either in GluN2B binding (I205K) or nucleotide binding (K43R). Using kinetic analysis of ATP interaction of both monomeric and heteromultimeric CaMKII mutants, we show that the reduction in S_0.5_ value of CaMKII for ATP as a result of GluN2B binding is independent of the holoenzyme structure. Contrary to what is observed with the wild type holoenzyme, V_max_ of monomeric CaMKII increased in the presence of GluN2B thereby resulting in a larger catalytic constant in comparison to phosphorylation of GluN2A. Taken together, it appears that the essential requirement for a change in the S_0.5_ value of a CaMKII subunit is the binding of GluN2B to the same subunit while any effect on the V_max_, due to GluN2B-binding, may additionally require the holoenzyme structure.

## Materials and Methods

### Materials

Bac-to-Bac baculovirus expression kit was from Gibco-BRL/Invitrogen, USA. QuikChange Site-Directed Mutagenesis Kit was from Stratagene, USA. BigDye Terminator v3.1 cycle sequencing kit was from Applied Biosystems, USA. Restriction and modification enzymes and other molecular biology related chemicals were from Promega, New England Biolabs or Amershampharmaciabiotec, USA. [γ-^32^P] ATP was from Bhabha Atomic Research Centre, India. Monoclonal antibody against the α-subunit of CaMKII and polyclonal antibody against His-tag were from Sigma Chemicals, USA. Anti-glutathione-S-transferase (GST) antibody was from Santacruz, USA. Anti-β-CaMKII monoclonal antibody was from Gibco-BRL, USA. *Sf*21 cells were from NCCS; India and High Five cells were from Invitrogen, USA.

### Construction of Association Domain Truncated Mutant of α-CaMKII

Site-directed mutagenesis was carried out using the QuikChange Site-Directed Mutagenesis Kit from Stratagene. The cDNA of α-CaMKII present in the expression vector pFastBac1-α-CaMKII that was used for expression of the wild-type (WT) enzyme served as the template to generate the mutant. A stop codon was introduced after the sequence encoding the catalytic domain in the CaMKII cDNA at 317^th^ aminoacid. The mutant protein upon expression had a molecular weight of around 34 kDa.

### Construction of the CaMKII Dual Mutant Vector

pFastBac^TM^Dual vector from Invitrogen was used for the construction. The cDNAs encoding α-I205K-CaMKII and β-K43R-CaMKII were used for the construction. α-I205K-CaMKII cDNA and β-K43R-CaMKII cDNA were cloned downstream to p10 and pH promoters respectively of the vector. The restriction sites utilized were *Xho*1 and *Kpn*1 for α-I205K-CaMKII and *Eco*R1 and *Hin*d III for β-K43R-CaMKII. The cDNAs of α-I205K-CaMKII and β-K43R-CaMKII were PCR amplified from donor vectors using primers incorporating the corresponding restriction sites and an N-terminal His-tag. Rest of the procedures was as per the instructions in the product manual for the dual vector. The bacmid encoding both the cDNAs was transfected into *Sf21* cells and was expressed in large scale in either *Sf21* or High Five insect cells. The expressed hetero-oligomer was shown to have both alpha and beta subunits with N-terminal His tags.

### Expression of GST Fusion Proteins of GluN2A and GluN2B in *E.coli*

**Figure 1:**
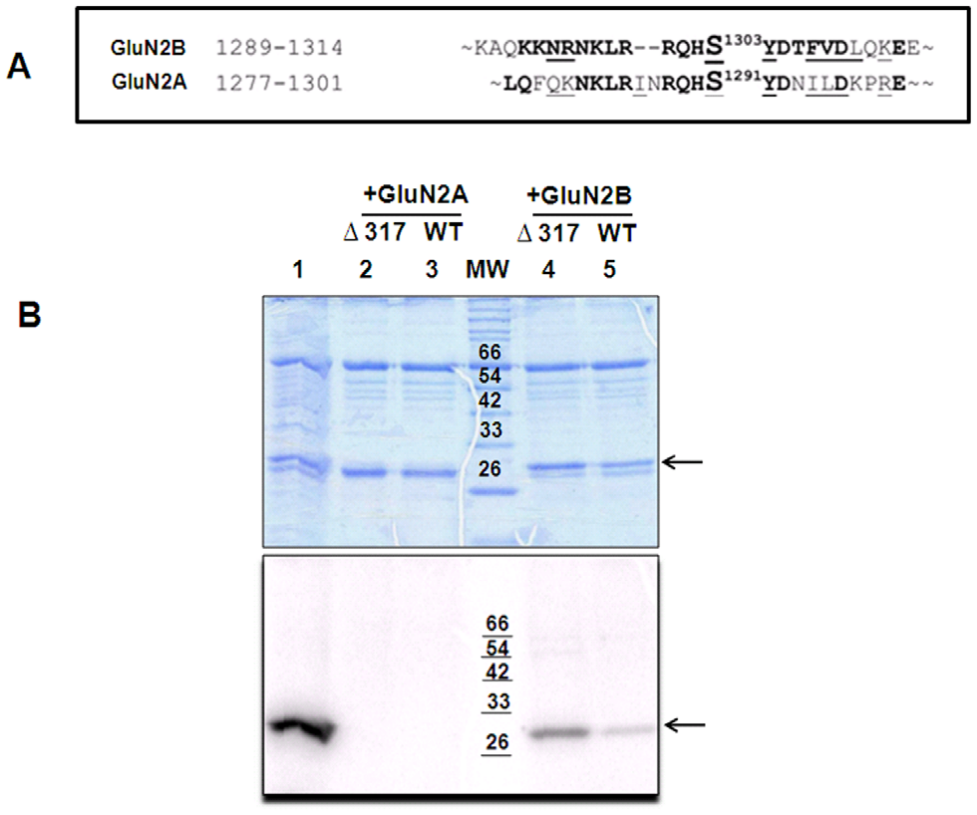
Monomeric CaMKII can bind to GluN2B sequence. A: Sequence alignment of GluN2A and GluN2B. The sequence motifs of GluN2A and GluN2B used for the study are aligned. The conserved residues are underlined with black lines and identical residues are shown in bold. The phosphorylatable residues are shown in a larger font. **B: GST-pull down assay followed by phosphorylation shows that monomeric CaMKII binds to GST-GluN2B.** GST-pull down assay was done with crude cell lysates of fusion proteins and monomeric CaMKII. Since western blotting could not detect the pulled down monomeric enzyme, we resorted to utilizing the calcium independent activity of the GluN2B bound CaMKII for detection. Upper panel shows the SDS-PAGE and lower panel shows the autoradiogram of the gel. Molecular weight marker positions in the autoradiogram are indicated with black lines. Phosphorylation has happened only in the reactions that have pulled down CaMKII. Arrow mark shows the position of GST-GluN2B. Lane 1: phosphorylation of GST-GluN2B (1271–1311) by WT-α-CaMKII before pull down; lanes 2 and 3: phosphorylation after pull down performed as negative control using GST-GluN2A (1265–1301) for Δ317-α-CaMKII and WT-α-CaMKII respectively; lane MW: Molecular weight marker. Molecular weights are indicated in kDa. Lanes 4 and 5: phosphorylation after pull down using GST-GluN2B (1271–1311) of Δ317-α-CaMKII and WT-α-CaMKII respectively. Data represents 2 experiments.

The fusion proteins, GST-GluN2A (GluN2A amino acid residues 1265–1301) and GST-GluN2B (GluN2B amino acid residues 1271–1311) were expressed in *E.coli* BL21 (DE3) using the respective cDNAs cloned in pGEX-2T vector as described earlier [14]. The sequence alignment of these two proteins is shown in Fig. 1A. The phosphorylation site as well as the CaMKII binding region of GluN2B is included in the presented sequence [7], [23].

### Preparation of Crude and Purified Forms of Wild Type, Truncated and Heteromeric CaMKII Expressed in Insect Cells

Preparation of crude insect cell lysate expressing α-CaMKII, and purified α-CaMKII were carried out as described before [14]. The expressed proteins were analyzed by western blot which showed a band of ∼52 kDa for the wild type α-CaMKII and a band of ∼34 kDa for the truncated α-CaMKII and bands of about 60 and 52 kDa corresponding to β and α-CaMKII respectively in the heteromeric mutant. For purification, the cells from three or more flasks (175 cc) were pooled and were resuspended in buffer containing 50 mM Pipes (pH 7.0), 5.0% betaine, 1 mM EGTA, 1 mM EDTA and 1X complete protease inhibitors cocktail (Sigma). The resuspended cells were lysed by homogenization using a Potter-Elvejham homogenizer (5 strokes) and sonication (3×10 s with 10 s intervals). The homogenate was clarified by centrifugation at 100,000 *g* for 30 min. The pellet obtained was rehomogenised in lysis buffer, centrifuged and the resulting supernatant was pooled with the first supernatant. The crude lysate thus obtained was loaded onto a phosphocellulose column, pre-equilibrated with equilibration buffer (50 mM Pipes, pH 7.0, 100 mM NaCl, 1 mM EGTA, 0.5 mM DTT and 1X protease inhibitor cocktail) and was incubated for 30 min. The bound proteins were eluted with 5 times the column volume (approx. 25 ml) of elution buffer (50 mM Pipes, pH 7.0, 500 mM NaCl, 1 mM EGTA, 0.5 mM DTT and 1x protease inhibitor cocktail). The eluate containing CaMKII activity was used for affinity purification on a CaM–Sepharose column. CaCl_2_ was added to a final concentration of 2 mM in the eluate. This was loaded onto CaM–Sepharose column (2–4 ml) pre-equilibrated with loading buffer [40 mM Hepes (pH 7.3), 2 mM CaCl_2_, 10% glycerol, 100 mM NaCl, 0.5 mM dithiothreitol, 0.1 mM PMSF]. The flow-through was reloaded and the column was washed with loading buffer containing 1 M NaCl. CaMKII was then eluted with buffer containing 40 mM Hepes (pH 7.3), 10% glycerol, 100 mM NaCl and 2.5 mM EGTA. The peak activity fractions were pooled and were used as the purified recombinant *α*-CaMKII.

### GST-Pull Down Assay

Glutathione sepharose 4B beads were washed thoroughly with PBS. Equal quantities of the beads were mixed separately with equal quantities of the crude lysates of each of the GST-fusion proteins in presence of Triton X-100 (0.1% final concentration). The mix was incubated in ice for 1 hr. The beads were then washed four times with PBS. Beads were separately incubated with equal concentrations of crude lysate of insect cells, expressing WT or mutant (monomeric or heteromultimeric) of CaMKII in binding buffer (50 mM PIPES, pH 7.0, 0.1% BSA, 150 mM NaCl, 0.1% Tween-20) with or without 1 mM CaCl_2_, 3 µM CaM for 1 hr at 4°C. Beads were then washed four times with PBS to remove unbound enzyme, resuspended in SDS-sample buffer and were kept in a boiling water bath for 3 min. The beads with bound complexes in SDS-sample buffer were then directly loaded onto a 10% gel and were subjected to western blotting using anti-His antibody and anti-GST antibody.

### Kinetic Analysis

GST fusion proteins of GluN2B and GluN2A were used as substrates for CaMKII in an *in vitro* radioactive assay as described before [14]. To determine the saturating concentration of the crude lysate or purified preparation of each of the fusion proteins separate experiments analyzing the saturation profiles of the fusion protein preparations were carried out. Each assay tube contained 50 mM Tris (pH 8.0), 10 mM MgCl_2_, 0.4 mM EGTA, 1.3 mM CaCl_2_, 5 µM CaM, 0.2 mg/ml BSA and saturating concentrations of either GST-GluN2A or GST-GluN2B as mentioned in Table 1. The concentration of ATP was varied from 1–400 µM. Assay was performed by first pre-incubating the fusion protein containing assay mixture for one minute at 30°C followed by a 1 minute phosphorylation reaction, initiated with the addition of CaMKII. Purified CaMKII was used for kinetic analyses. The quantities of the enzyme used were 0.01–0.017 µg for WT-CaMKII, 0.15 µg for Δ317-α-CaMKII and 1.9 µg for heteromeric CaMKII. The reaction was stopped by the addition of 5 µl of 5X SDS sample buffer and the samples were run on an SDS-PAGE. After the staining and destaining processes, the gels were dried and were exposed to a phosphor screen. The screen was later scanned in a Phosphor Imager and the gel bands in the autoradiogram corresponding to phosphorylated bands were quantitated using QuantityOne software from Bio-Rad. The data were analysed using Eadie-Hofstee and Hill’s plot. The final values for V_max_, S_0.5,_ Hill coefficient, etc. are presented as mean ± standard deviation of data from at least 3 independent experiments. The p values were calculated using student’s t test.

**Table 1:** Kinetic parameters of WT, Monomeric and Heteromultimeric CaMKII for ATP.

Enzyme used	WT-α-CaMKII		Δ317 CaMKII		Heteromeric CaMKII	
Substrate	GluN2A	GluN2B	GluN2A	GluN2B	GluN2A	GluN2B
**S_0.5_ µM**	68±5	5.9±1.3*	156±14	57±0.7*	13±2.2	16±1.4
**V_max_ µmoles. min^−1^.mg protein^−1^**	0.176±0.027	0.022±0.003*	0.015±0.0007	0.024±0.006*	0.0032±0.001	0.0034±0.0006
**V_max_/S_0.5_** (×10**^−^** ^3^)	2.6±0.2	3.7±0.4	0.09±0.01	0.4±0.099*	0.24±0.064	0.22±0.045
**Hill Coefficient**	1.93±0.31	1.18±0.3*	0.98±0.07	1.17±0.16	1.2±0.11	1.0±0.07

The ATP kinetic parameters of WT-CaMKII, Δ317-α-CaMKII and heteromeric (α-I205K)-(β-K43R) CaMKII are given. The quantity of CaMKII used for the assays was: WT-CaMKII-0.01–0.017 µg; Monomeric Δ317-α-CaMKII- (0.15 µg); Heteromeric (α-I205K)-(β-K43R) CaMKII-1.9 µg. For heteromeric CaMKII assay, saturating concentrations of purified preparations of GST-GluN2A (6.5 µM) and GST-GluN2B (9.4 µM) were used. For the rest of the assays saturating concentrations of fusion proteins in bacterial lysates were used. p values were calculated in comparison with corresponding GluN2A data in each set of experiments. p<0.05 was considered significant which are indicated with asterisk (*). Representative data used for estimation of kinetic parameters is shown in Fig. S1.

### Phosphorylation of the Pull Down Complex of CaMKII and GST-GluN2B

GST-pull down assay was used to show that GluN2B can bind CaMKII even when the enzyme is in its monomeric form. A control reaction with GST-GluN2A was also run simultaneously. The complex obtained after the GST-pull down assay was subjected to phosphorylation in the absence of CaCl_2_ to detect the activity of the GST-GluN2B bound CaMKII. The reaction conditions employed for fusion protein phosphorylation as described in the previous section were used, with the exception that the EGTA concentration was at least 0.5 mM higher than the concentration of Ca^2+^ that was added during the pull down binding reaction and concentration of ATP was 200 µM.

## Results

### CaMKII Monomers can Bind to GST-GluN2B and not GST-GluN2A

To understand whether the binding of CaMKII to GluN2B is dependent on the oligomeric structure of CaMKII, we subjected the truncated monomeric α-CaMKII (Δ317-α-CaMKII) to GST pull down assay. Since western blotting could not detect the pulled down monomeric enzyme [22], we resorted to utilizing the calcium independent activity of the GluN2B bound CaMKII for detection (Fig.1B). We assume that the amount of pulled down truncated enzyme did not present sufficient antigenic sites to permit antibody detection by western blot. We were able to detect calcium independent activity in the wild type as well as Δ317-α-CaMKII samples, pulled down using GST-GluN2B. This shows that GluN2B specifically binds CaMKII even when the enzyme is in its monomeric form. Control reactions with GST-GluN2A did not show phosphorylation activity because of the absence of any pulled down CaMKII.

### ATP Kinetic Parameters for Monomeric CaMKII are Altered When GST-GluN2B was the Protein Substrate

Using enzyme kinetics, we show that GluN2B can modulate the interaction of ATP with monomeric CaMKII. We determined the kinetic parameters of ATP substrate with either GST-GluN2B or GST-GluN2A as protein substrates. Kinetic analyses were carried out for both WT holoenzyme of CaMKII as well as truncated monomeric CaMKII (Table 1). The S_0.5_ value for ATP was significantly lower with GST-GluN2B when compared with that obtained with GST-GluN2A for both forms of the enzyme (Table 1). Although the magnitudes of the kinetic parameters were different for the two forms of CaMKII, a reduction in the S_0.5_ values for ATP in presence of GST-GluN2B was observed in both the enzyme forms. Interestingly, monomeric CaMKII yielded a ∼4 fold higher value for the apparent catalytic constant, V_max_/S_0.5,_ in the presence of GST-GluN2B compared to the value obtained in presence of GST-GluN2A. The WT-CaMKII did not show any change between GluN2B and GluN2A sequences for the catalytic constant. This was mainly because of the increased V_max_ value of the monomeric enzyme in the presence of GST-GluN2B while for WT-CaMKII, the V_max_ was reduced with GST-GluN2B. The Hill co-efficient value which is the cooperativity index was nearly 2 for WT-CaMKII with GST-GluN2A while it was close to 1 with GST-GluN2B as reported before [14]. This showed that the catalytic site binding of ATP, which was cooperative became non-cooperative in the presence of GST-GluN2B. Monomeric CaMKII showed Hill co-efficient value of around 1 in both the cases as expected (Table 1).

### Expression and GST Pull Down Assay of Heteromeric CaMKII

**Figure 2:**
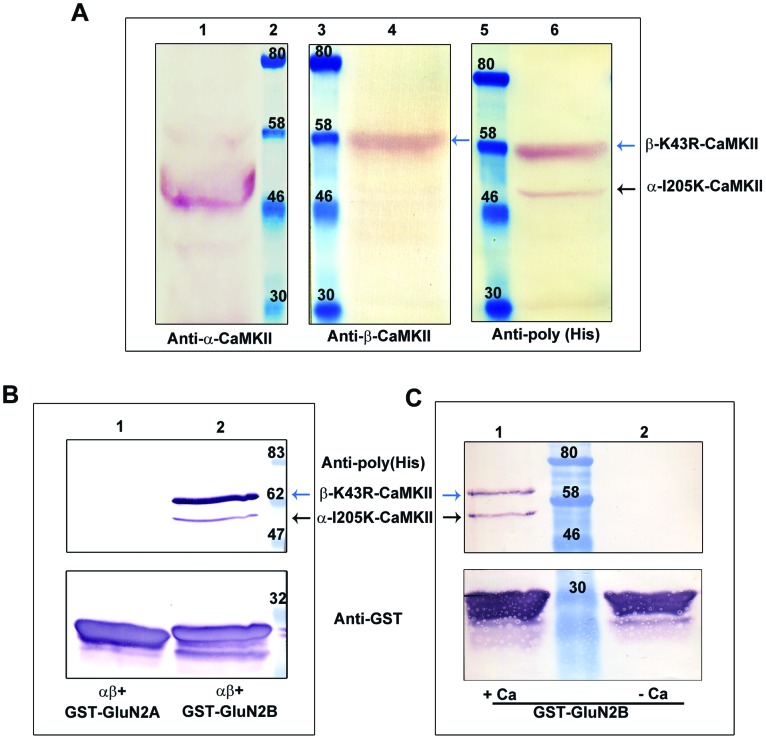
GST-pull down assay shows that α-I205K-CaMKII and β-K43R-CaMKII can form heteromultimers. The mutants, α-I205K-CaMKII and β-K43R-CaMKII, when expressed from a single vector, form heteromultimers and are pulled down by GST-GluN2B. **A: Western blot of (α-I205K)-(β-K43R)-CaMKII expressed in insect cell line.** The heteromeric CaMKII was prepared by coexpressing GluN2B binding defective α-subunits (I205K) with β-subunit defective in nucleotide binding (K43R) using pFastBac^TM^Dual vector. Both the CaMKII mutants were His-tagged at their N-terminii. Lane 2, 3 and 5: Molecular weight marker; Lane 1: Lysate (50 µg) expressing heteromer probed with anti-α-CaMKII antibody; Lane 4: Lysate (50 µg) expressing heteromer probed with anti-β-CaMKII antibody; Lane 6: Lysate (50 µg) expressing heteromer probed with anti-(poly) His antibody. The arrows indicate the positions of α and β subunits in each lane. **B: Heteromeric CaMKII binds specifically to GST-GluN2B.** Western blot following GST pull down assay of the heteromeric CaMKII mutant is shown. GST-Pull down assays were done with crude cell lysates of fusion proteins and heteromeric CaMKII. Upper panel was probed with anti-(poly) His antibody and lower panel was probed with anti-GST antibody. Lane 1: Pull down with GST-GluN2A; Lane 2: Pull down with GST-GluN2B. Data represents 4 experiments. **C: GluN2B binding of heteromeric CaMKII is calcium dependent.** Western blot following GST pull down assay of the heteromeric CaMKII mutant with GST- GluN2B is shown. GST-Pull down assays were done with crude cell lysates of fusion proteins and heteromeric CaMKII. Upper panel was probed with anti-(poly) His Antibody and lower panel was probed with anti- GST antibody. Lane 1: Pull down in presence of Ca^2+^; Lane 2: Pull down in the absence of Ca^2+^. Data represents 3 experiments.

We made a heteromeric CaMKII [(α-I205K)-(β-K43R)-CaMKII] by coexpressing GluN2B binding defective subunits (I205K) with subunits defective in nucleotide binding (K43R) [24], [25]. pFastBac^TM^Dual vector was used to clone the cDNAs of α-I205K-CaMKII and β-K43R-CaMKII to yield the final construct of pFastBac Dual-(α-I205K)-(β-K43R)-CaMKII in which both subunits had N-terminal His-tags. The kinase-dead mutant (K43R) of CaMKII [24], [26], [27] can bind to GluN2B, but cannot bind ATP and hence would not contribute to kinetic parameters of ATP. The activity of the heteromeric CaMKII is due to α-I205K-CaMKII which in turn does not bind GluN2B [10], [12], [14], [22], [28]. This construct was expressed in High Five insect cells and the lysate was used to check the level of expression of the heteromeric enzyme. Same quantity of cell lysate was loaded into different wells of an SDS-PAGE to probe with different antibodies. The subunits were detected by western blot either separately with anti-α-CaMKII and anti-β-CaMKII antibodies or with anti-His tag antibody since both subunits carried His-tags (Fig. 2A). Insect cell lysate expressing the heteromeric CaMKII was used for GST-pull down assay and we show that the heteromeric CaMKII can bind GluN2B, but not GluN2A, in a Ca^2+^ dependent manner (Fig. 2B & 2C). The pulled down enzyme complex shows the presence of α-I205K-subunit that is defective in binding to GluN2B showing that the two mutant subunits indeed form a heteromultimer. Moreover, the ratios between the quantities of the subunits (beta/alpha) in the crude lysate (2.1±0.7) and in the pull down complex (2.8±1.3) were similar. This suggests that the enzymes in the crude lysate as well as in the pull-down complex are predominantly heteromers.

### ATP Saturation Kinetics of Heteromeric (α-I205K)-(β-K43R)-CaMKII is not Altered by GluN2B

The kinetic parameters obtained for heteromeric CaMKII showed similar values for both GST-GluN2A and GST-GluN2B (Table 1). The S_0.5_ and V_max_ values were more or less similar in the presence of both the protein substrates. The Hill co-efficient was close to 1 showing absence of ATP cooperativity in both cases. The apparent catalytic constants of V_max_/S_0.5_ for both of the protein substrates were also similar.

## Discussion

In the current study, we carried out kinetic analyses to see the effect of GluN2B on ATP substrate interaction in the association domain truncated monomeric CaMKII and compared it to the WT holoenzyme of CaMKII which is known to exist as a dodecamer [29]. To validate our findings, we also studied the ATP kinetics of a heteromultimeric CaMKII having GluN2B binding defective (α-I205K-CaMKII) mutant subunits interspersed between ATP binding impaired (β-K43R-CaMKII) mutant subunits.

Our earlier result showing that GluN2B binding abolishes cooperativity as well as reduces V_max_ in the CaMKII holoenzyme [14] was observed in our current experiments as well (Table 1). For the monomeric CaMKII also, we observed that there is an alteration in the kinetic parameters for the ATP substrate in the presence of GST-GluN2B (Table 1). There was a ∼3 fold reduction of S_0.5_ in the presence of GluN2B similar to the WT CaMKII holoenzyme which showed about 11 fold reduction. This implied that both the enzyme forms show increased affinity for ATP in the presence of GluN2B (Table 1) [14], [15]. However, the V_max_ of monomeric CaMKII increased by about 2 fold in the presence of GST-GluN2B which is contrary to what was observed in the WT-multimeric CaMKII. This increase in activity along with the decrease in S_0.5_ value for ATP of monomeric CaMKII thus resulted in an approximately 4.0 fold higher V_max_/S_0.5_ value in the presence of GST-GluN2B as compared to the presence of GST-GluN2A. Such an increase in the V_max_/S_0.5_ value did not occur in the multimeric WT-CaMKII. It appears that the GluN2B-induced structural changes are significantly different between the monomeric and multimeric forms of the enzyme. This implies that the multimeric structure does play a role in the modulation of ATP kinetics of WT-CaMKII induced by GluN2B. The Hill co-efficient value observed for monomeric Δ317-α-CaMKII was around 1 with either GST-GluN2A or GST-GluN2B since cooperativity requires multimeric structure.

In order to validate our findings with the monomeric CaMKII and to investigate the possibility of an inter-subunit mechanism in the GluN2B mediated modulation in a CaMKII multimer, we utilized the heteromultimeric CaMKII. The heteromultimer [(α-I205K)-(β-K43R)-CaMKII] carried mutant subunits in which the kinase active subunits were unable to bind GluN2B (I205K mutation) while the kinase inactive subunits (K43R mutation) can bind GluN2B. In this case the kinetics would be a reflection of the activity of the non-binding subunits. The kinetics would have been affected had the GluN2B bound CaMKII subunits transmitted any modulatory changes to the adjacent active subunits too. But we observe that the GluN2B induced changes in S_0.5_ and V_max_ parameters of ATP kinetics that were evident in the WT enzyme were clearly absent in the heteromeric enzyme (Table 1). The Hill co-efficient for heteromeric CaMKII was close to 1 with both GluN2A and GluN2B substrates, which was expected, because the K43R subunit being ATP binding defective curtails the cooperative interactions between the subunits. The other kinetic parameters obtained were similar in the presence of both GluN2A as well as GluN2B which suggests that for the GluN2B mediated effect to take place, binding has to happen to individual subunits and that the binding to adjacent subunits is not sufficient to alter kinetics. The kinetic analyses of heteromeric CaMKII thus substantiate our observations on the monomeric CaMKII which suggest that a major component of the GluN2B mediated catalytic modulation is restricted to the CaMKII subunit involved in the binding.

Physiological relevance of the GluN2B-mediated alteration of the kinetics of CaMKII needs further experimental support. However, GluN2B-induced reduction in V_max_ value would likely be a regulatory mechanism to occur at the millimolar levels of ATP present physiologically since GluN2B bound CaMKII has been shown to get saturated at low concentrations of ATP (14, 15). Although it is difficult to predict the physiological relevance of the micromolar level changes in S_0.5_ values, in the light of recent reports that suggest the existence of ATP concentration gradients in synaptic compartments [30] with ATP being depleted rapidly near the synaptic membranes, it may well be considered possible that the GluN2B-induced changes in S_0.5_ values at micromolar levels would also have physiological significance.

Overall, the data presented in this study suggests that structural changes within a subunit of CaMKII are necessary for mediating the GluN2B-induced regulation and emphasizes the role of the multimeric structure in effecting such a regulation.

## Supporting Information

Figure S1 **ATP saturation profiles differ among WT, monomeric and heteromeric forms of CaMKII.** ATP saturation profiles of phosphorylation of GluN2A and GluN2B by WT (A and B), Δ317 (C and D) and Heteromer (E and F) are shown from representative experiments. Three or more such experiments were used in each case to obtain the kinetic parameters shown in Table 1.
(TIF)
