# Supplementary figures and images for: Effect of Multimeric Structure of CaMKII in the GluN2B-Mediated Modulation of Kinetic Parameters of ATP
Source: PLoS One. 2012 Sep 18;7(9):e45064. doi: 10.1371/journal.pone.0045064 (PMC3445591; doi:10.1371/journal.pone.0045064)

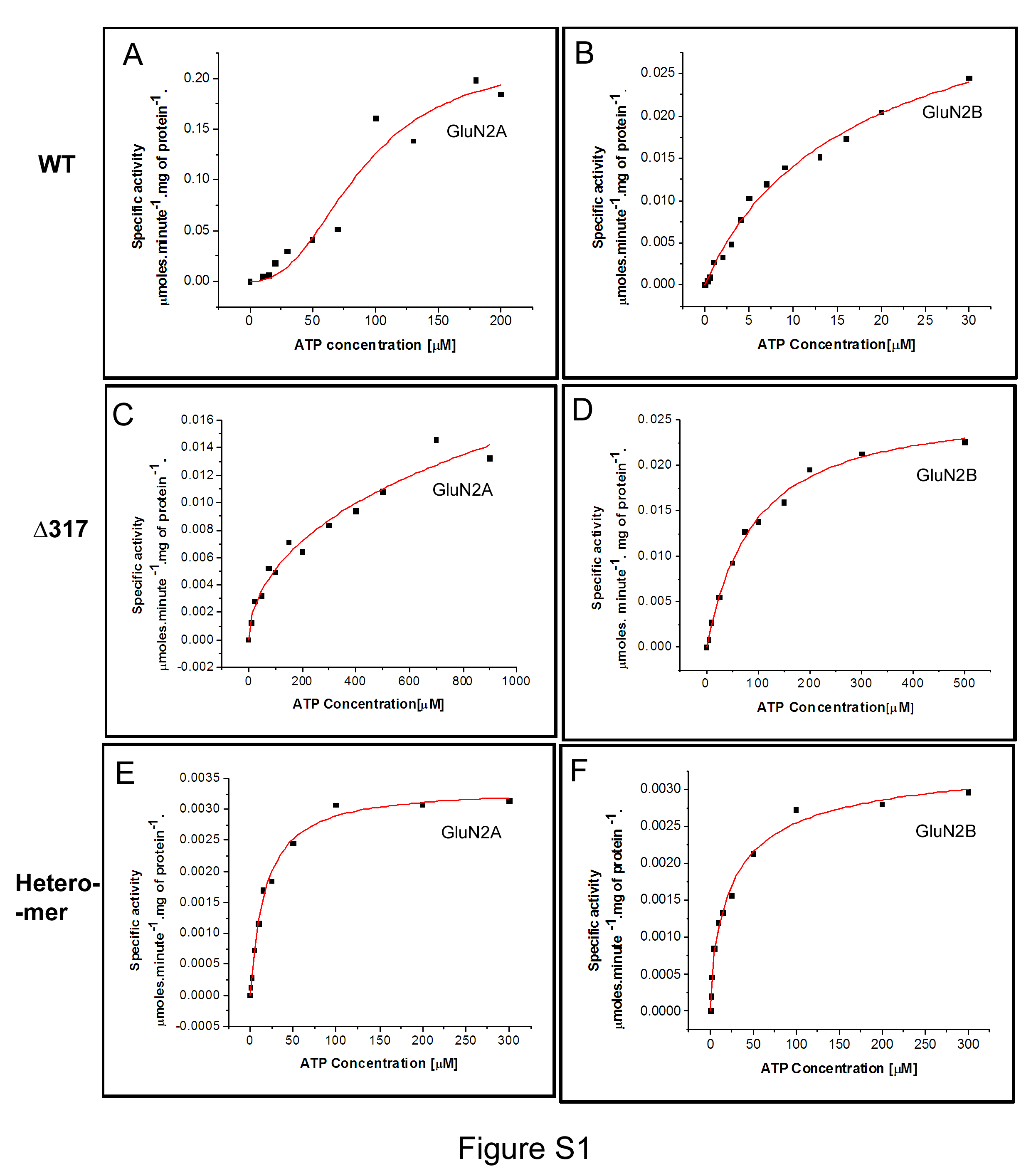

Supplement: Figure S1 — ATP saturation profiles differ among WT, monomeric and heteromeric forms of CaMKII. ATP saturation profiles of phosphorylation of GluN2A and GluN2B by WT (A and B), Δ317 (C and D) and Heteromer (E and F) are shown from representative experiments. Three or more such experiments were used in each case to obtain the kinetic parameters shown in Table 1. (TIF) [file pone.0045064.s001.tif]
